# Growth under Fluctuating Light Reveals Large Trait Variation in a Panel of Arabidopsis Accessions

**DOI:** 10.3390/plants9030316

**Published:** 2020-03-03

**Authors:** Elias Kaiser, Dirk Walther, Ute Armbruster

**Affiliations:** 1 Max Planck Institute of Molecular Plant Physiology, Wissenschaftspark Golm, Am Mühlenberg 1, 14476 Potsdam, Germany; walther@mpimp-golm.mpg.de; 2 Horticulture and Product Physiology, Wageningen University, Droevendaalsesteeg 1, 6708 PB Wageningen, The Netherlands

**Keywords:** acclimation, chlorophyll *a* fluorescence, fluctuating light, natural variation, photosynthesis

## Abstract

The capacity of photoautotrophs to fix carbon depends on the efficiency of the conversion of light energy into chemical potential by photosynthesis. In nature, light input into photosynthesis can change very rapidly and dramatically. To analyze how genetic variation in *Arabidopsis thaliana* affects photosynthesis and growth under dynamic light conditions, 36 randomly chosen natural accessions were grown under uniform and fluctuating light intensities. After 14 days of growth under uniform or fluctuating light regimes, maximum photosystem II quantum efficiency (F_v_/F_m_) was determined, photosystem II operating efficiency (Φ_PSII_) and non-photochemical quenching (NPQ) were measured in low light, and projected leaf area (PLA) as well as the number of visible leaves were estimated. Our data show that Φ_PSII_ and PLA were decreased and NPQ was increased, while F_v_/F_m_ and number of visible leaves were unaffected, in most accessions grown under fluctuating compared to uniform light. There were large changes between accessions for most of these parameters, which, however, were not correlated with genomic variation. Fast growing accessions under uniform light showed the largest growth reductions under fluctuating light, which correlated strongly with a reduction in Φ_PSII_, suggesting that, under fluctuating light, photosynthesis controls growth and not vice versa.

## 1. Introduction

In nature, light energy supply for plant photosynthesis varies strongly in both amplitude and frequency. How plants respond to dynamic light environments is still poorly understood. This has many reasons, among them being that for most experiments, plants are grown under standard, highly controlled, and uniform light regimes (U). However, plant responses strongly depend on the environment that plants have acclimated to, as they adjust their metabolism to cope most efficiently with the prevailing condition. Several recent studies focused on the model plant *Arabidopsis thaliana* (hereafter: Arabidopsis) under dynamic light [1,2,3], but these were restricted to few accessions only and thus did not assess the effects of genetic factors. The study of a larger set of genotypes is warranted, also because photosynthesis shows great intraspecific variation in Arabidopsis [4,5], and variation on the genetic level has been shown to translate more strongly into phenotypic variation under fluctuating light regimes (FL; [6]). Using genome-wide association mapping, natural variation in the photosynthetic response to high light could be linked to several quantitative trait loci [5], supporting the notion that in Arabidopsis, traits linked to photosynthesis are heritable.

Compared to U, FL with the same average intensity often reduces plant growth [7,8,9,10], although exceptions exist [11]. There are at least two reasons for this reduction. Firstly, photosynthesis responds nonlinearly to light, i.e., at higher light intensities the rate of photosynthesis is limited by its capacity for CO_2_ fixation, in turn leading to the activation of photoprotective mechanisms that dissipate absorbed light energy as heat, and resulting in a decrease in photosynthetic efficiency (reviewed by [12]). Leaves under FL, in contrast to U, are generally exposed to light periods during which photosynthesis is saturated. Secondly, photosynthesis generally lags behind rapid changes in light intensity, but the loss in CO_2_ fixation after an increase in light intensity typically exceeds any gains in CO_2_ fixation after a decrease (though see [13] for a further discussion on the role of post-illumination CO_2_ fixation). The latter is partially connected to the slow relaxation of photoprotective mechanisms in low light [14].

Theoretically, plant growth is linked to how quickly photosynthesis can switch between protective mechanisms in high light and highly efficient light capture and conversion in shade periods [14]. Insufficient protection in high light causes photooxidative damage [15], while overprotection can result in low rates of photosynthesis through a reduction in the operating efficiency of PSII (Φ_PSII_), particularly in shade periods when light availability limits the rate of photosynthesis [16]. Non-photochemical quenching (NPQ) is a central photoprotective mechanism in plants [17]. Most NPQ is rapidly reversible, and is controlled by the proton concentration of the lumen (reviewed by [12]). Light-driven electron transport from water to NADPH along the thylakoid localized electron transport chain is coupled to the transfer of protons from the stroma into the lumen. Protons then exit the lumen via the ATP synthase, thereby providing the energy required for ATP synthesis. In high light conditions, when downstream metabolic reactions are limiting, the proton concentration in the lumen rises, as efflux via the ATP synthase is restricted [18,19]. Above a threshold, the proton concentration in the lumen induces a reorganization of the PSII supercomplex via protonating key amino acid residues of the PsbS protein and activates the violaxanthin-deepoxidase (VDE), both of which are important for maximum pH-dependent quenching, which is also referred to as energy-dependent quenching (qE, reviewed by [12]). Under prolonged stress conditions, photoinhibitory quenching (qI) is induced, which coincides with oxidative damage to the D1 protein of photosystem II [17]. Such damage also causes an increased Chl *a* fluorescence of dark acclimated plants and is reflected as a decrease in F_v_/F_m_, which is a measure of the maximum quantum efficiency of PSII. However, reductions of F_v_/F_m_ are often only observed under relatively harsh conditions, whereas NPQ and Φ_PSII_ already respond to milder stresses.

While the rapid response of photosynthesis to high light is comparably well studied on the molecular level (reviewed by [12]), much less is known about molecular mechanisms that allow photosynthesis to rapidly adjust to low light periods. Only recently, it was shown that plants contain at least one molecular player that accelerates the response of photosynthesis to shade periods [16,19]. Furthermore, the capacity for NPQ can be upregulated in Arabidopsis acclimated to FL [1,20], but it is not known whether (i) such an upregulation might reduce Φ_PSII_, particularly in shade periods, during which this reduction could reduce photosynthetic efficiency and growth and (ii) whether natural genetic variation exists for these phenomena. We hypothesized that (i) NPQ would be upregulated and Φ_PSII_ would be decreased in low light in plants grown under FL compared to U, (ii) dark-adapted F_v_/F_m_ would be largely unaffected by FL relative to U, (iii) growth would be reduced under FL compared to U, and that (iv) large differences for the extent of these changes between FL and U would become apparent between genotypes. To test these hypotheses, we grew 36 natural accessions of Arabidopsis (Table 1) under U and FL and next to their growth and development assessed Φ_PSII_ and NPQ under low light, as well as dark-adapted F_v_/F_m_.

## 2. Results

### 2.1. Chlorophyll a Fluorescence

**Figure 1:**
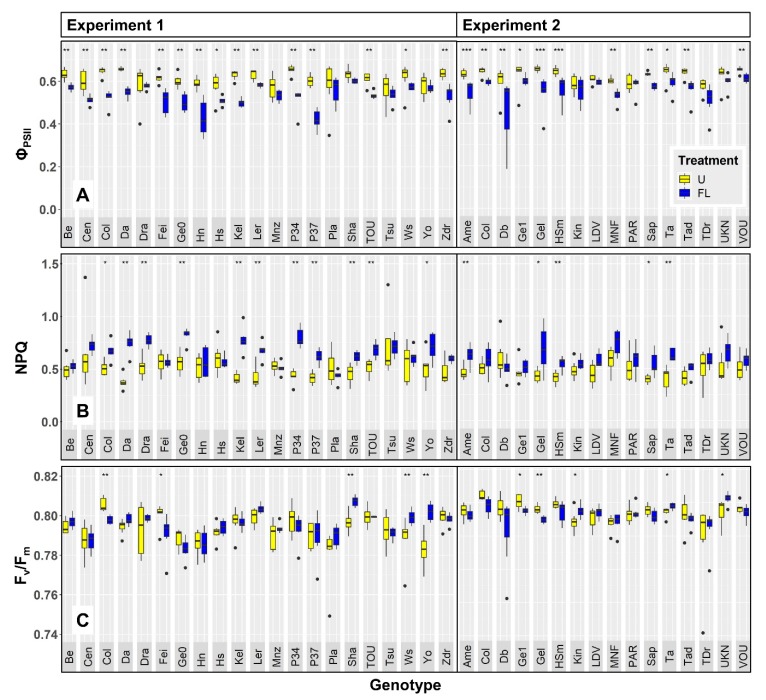
Chlorophyll *a* fluorescence analysis of 36 Arabidopsis accessions acclimated to uniform (U; yellow boxes) and fluctuating light intensities (FL; blue boxes), grown in two different experiments. (**A**) photosystem II operating efficiency (Φ_PSII_), (**B**) non-photochemical quenching (NPQ), and (**C**) photosystem II maximum quantum efficiency (F_v_/F_m_). Φ_PSII_ and NPQ were measured at 90 µmol m^−2^ s^−1^, whereas F_v_/F_m_ was measured on dark-adapted leaves. Bars depict interquartile range (IQR; 25th–75th percentile) and median (thick line inside bar), whiskers depict data up to 1.5 × IQR, dots outside whiskers depict outliers (>1.5 × IQR). In the case of significant differences between average values under U and FL, these are shown for a given accession as: *** = *p* < 0.001, ** = *p* < 0.01 and * = *p* < 0.05 (n = 5–7).

Twenty-six accessions (i.e., 72%) showed significantly reduced values of Φ_PSII_ when grown under FL as compared to growth under U (Figure 1A). No accession showed increased Φ_PSII_ under FL. NPQ was significantly increased in 13 (36%) of the FL-grown accessions (Figure 1B), and none of the FL-grown accessions showed significantly reduced NPQ. Maximum quantum efficiency of photosystem II (F_v_/F_m_), measured in dark-adapted leaves, showed a very different pattern compared to Φ_PSII_ and NPQ: of the ten accessions showing a significant effect of FL on F_v_/F_m_, six FL-grown accessions showed significantly enhanced F_v_/F_m_, while four others showed significantly reduced F_v_/F_m_ (Figure 1C).

### 2.2. Growth and Development

**Figure 2:**
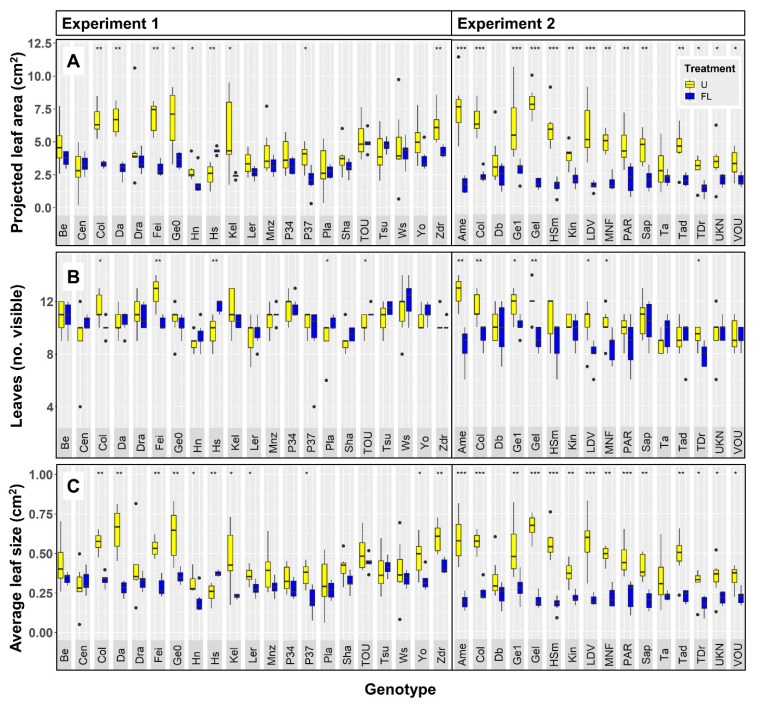
Growth and development of 36 Arabidopsis accessions acclimated to uniform (U; yellow boxes) and fluctuating light intensities (FL; blue boxes), grown in two different experiments. (**A**) Projected leaf area, (**B**) number of visible leaves and (**C**) average leaf size. Values were obtained from chlorophyll *a* fluorescence pictures. Bars depict interquartile range (IQR; 25th–75th percentile) and median (thick line inside bar), whiskers depict data up to 1.5 × IQR, dots outside whiskers depict outliers (>1.5 × IQR). In the case of significant differences between average values under U and FL, these are shown for a given accession as: *** = *p* < 0.001, ** = *p* < 0.01 and * = *p* < 0.05 (n = 5–7).

Projected leaf area (PLA) and the number of visible leaves were assessed as proxies for growth and development, respectively. PLA of plants was significantly reduced when grown under FL as compared to U in 22 (61%) accessions (Figure 2A). On average, PLA was reduced by 36% across all accessions, meaning that the reduction in PLA was not only significant for many accessions, but also substantial (although PLA showed a large coefficient of variation: 42.3%). However, one accession, Hs-0, had a strongly increased PLA (74%) under FL compared to U (Figure 2A). Of the 12 accessions that showed significant treatment effects on the number of visible leaves, FL decreased leaf number in nine cases, but increased it in another three cases (among which was Hs-0; Figure 2B). Finally, to account for the possibility that differences in projected leaf area may be caused by differences in the number of leaves, average leaf size was determined by dividing projected leaf area by leaf number (Figure 2C). When expressed this way, 24 (67%) FL-grown accessions showed significant reductions in average leaf size, most of which overlapped with those showing reduced PLA. Hs-0 again displayed a significant increase in average leaf size when grown under FL. Two other accessions, Tsu-0 and Cen-0, showed larger growth- and development-related parameters under FL, albeit to a lesser extent than Hs-0. While for Tsu-0 this did not correlate with treatment effects on Chl *a* fluorescence data, Cen-0, like Hs-0, showed positive growth and development despite reduced Φ_PSII_ and increased NPQ in FL as compared to U (Figure 1 and Figure 2). Accession Col-0 had been grown in both experiments: Φ_PSII_, leaf area, leaf number and average leaf area were all significantly reduced in FL-grown Col-0, compared to Col-0 under uniform irradiance, in both experiments (Figure 1 and Figure 2). NPQ was significantly increased under FL in Col-0 in Exp. 1 but not in Exp. 2, although data showed the same tendency (Figure 1B). Similarly, F_v_/F_m_ was significantly reduced in FL-treated Col-0 in Exp. 1 but not in Exp. 2, however it tended to be reduced (Figure 1C). Altogether, these data suggest that similar conclusions could be drawn from both FL experiments, reassuring us of the repeatability of the experimental setup used.

We observed a large trait variation across accessions and treatments (Figure 1 and Figure 2). For example, projected leaf area for a given accession ranged from 1.4 to 8.0 cm^2^ (Figure 2A), resulting in a coefficient of variation (CV) of 42.3%. Average leaf size varied to a slightly lesser degree (35.9% CV; Figure 2C), while leaf number per plant displayed a comparably low CV of 10.3% (Figure 2B). Of the chlorophyll *a* fluorescence traits, NPQ showed the largest variation (0.38–0.82; 19.2% CV; Figure 1B) while Φ_PSII_ (0.42–0.66; 9.0% CV; Figure 1A) varied comparably less. In contrast to these values, F_v_/F_m_ showed a very small variation of only 0.8% CV (Figure 1C).

### 2.3. Principle Component Analysis

**Figure 3:**
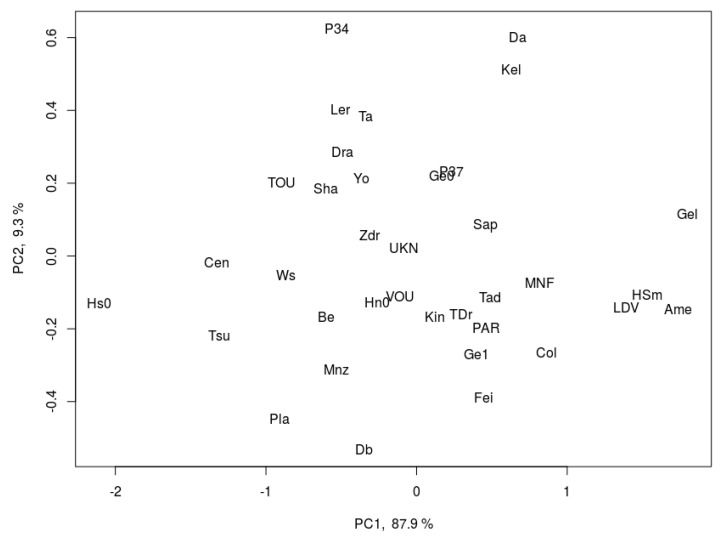
Principal component analysis of 36 Arabidopsis accessions, based on differences in phenotypical response ratio under fluctuating vs. uniform light (log−2 transformed FL/U ratio). PC1 accounts for 87.9% and PC2 accounts for 9.3% of the total variation.

For each trait and accession, we calculated a response ratio by dividing the value of the trait due to growth under FL by its value under U. Based on the log−2 transformed response ratios of all phenotypes to FL relative to U, a principal component analysis was constructed (Figure 3). Principal component 1 accounted for 87.9% of the total variance, and was characterized by large loadings for traits in growth and development (PLA, leaf size, leaf number). Principal component 2 accounted for another 9.3%, and was dominated by a large loading for NPQ. Given that principal component 1 accounted for most of the variation and was dominated by traits related to growth, the PCA reveals that variation in growth was the biggest determinant for the overall variation in our data. Generally, there was little clustering of accessions based on any of the two principal components (Figure 3), suggesting large genetic variation for both groups of traits among the 36 accessions studied here.

### 2.4. Phenotypic and Genomic Cluster Analysis

**Figure 4:**
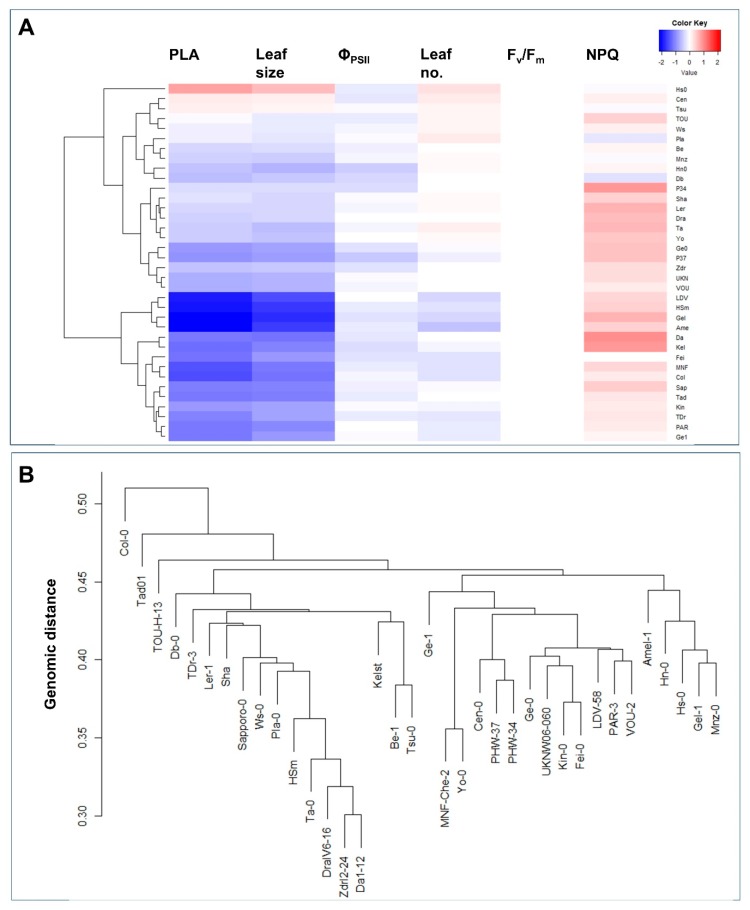
Clustering of 36 Arabidopsis accessions based on (**A**) differences in phenotypical logarithmic response ratios under fluctuating vs. uniform light (FL/U ratio; plot produced using heatmap.2 function with default settings of R package gplot) and (**B**) genomic distances, based on published SNP data (single linkage).

Accessions were clustered based on values of the log−2 transformed response ratios (Figure 4A). Accessions were most strongly divided into different groups based on growth (PLA, leaf size), with Hs-0 being distinct from all other accessions. Another group of accessions, LDV-58, HSm, Gel-1, and Amel-1 could be defined as FL-sensitive in that these accessions showed the strongest reductions in growth and number of leaves, while also showing some reductions in Φ_PSII_ and relatively large increases in NPQ (Figure 4A). When clustered based on their genomic differences, on the other hand, accessions showed an entirely different pattern (Figure 4B). For example, Col-0 was suggested to have the largest genomic distance from all other accessions, while phenotypic clustering suggested its response to FL to be close to that of, e.g., Fei-0 and MNF-Che-2 (Figure 4A). Another example for the incongruence between genomic and phenotypic clustering is a group of closely related accessions that all originated in the Czech Republic: HSm, Ta-0, DraIV6-16, ZdrI2-24 and Da(1)-12 (Figure 4B, Table 1). The phenotypic analysis, on the other hand, did not indicate a close link between these accessions (Figure 4A). Indeed, no significant correlation was found between log−2 transformed response ratios and corresponding genomic distances between all pairwise comparisons among the 36 accessions (36 × 35/2 pairs; Pearson correlation coefficient = −0.02; *p* = 0.56). Together, these results suggest that the genomic distance alone cannot be used to predict behavior under FL compared to U, indicating that genetic variation in specific genes may account for the difference.

### 2.5. Correlation Analysis

**Figure 5:**
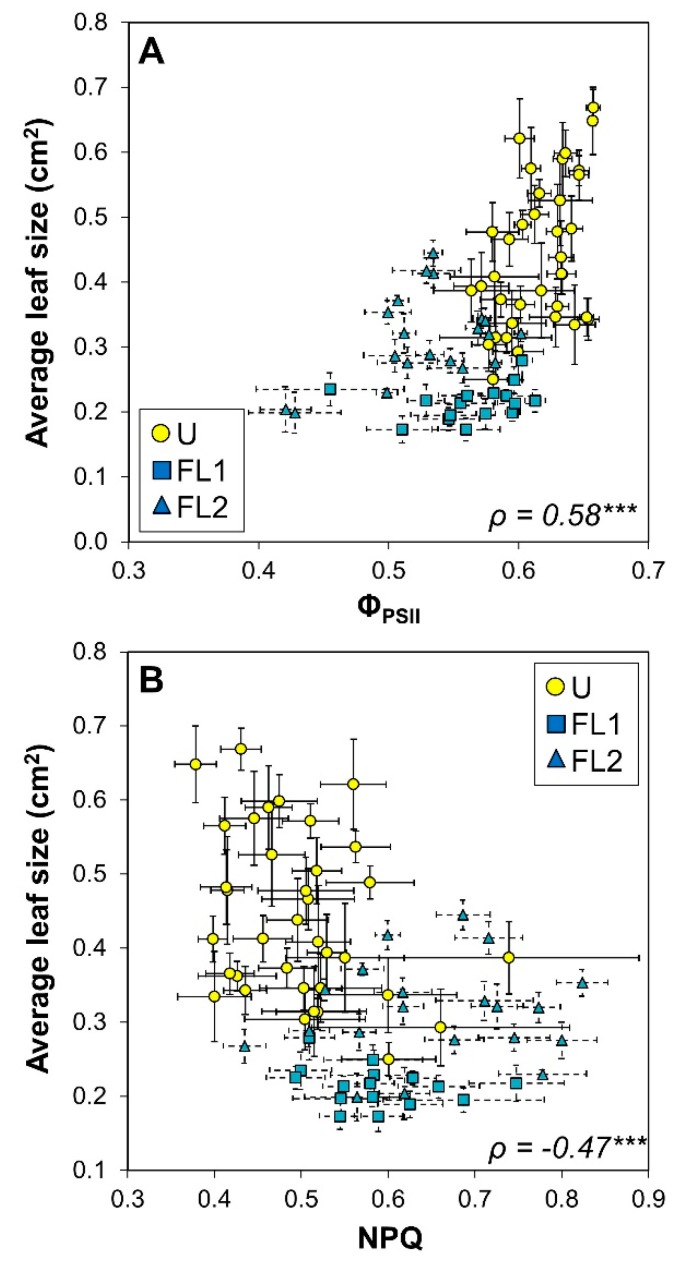
Relationships between average leaf size and (**A**) photosystem II operating efficiency (Φ_PSII_) and (**B**) non-photochemical quenching (NPQ) in 36 Arabidopsis accessions acclimated to uniform (U) and fluctuating light intensities (FL). Data from plants grown under FL are sorted by experiment 1 (squares) and experiment 2 (triangles). Averages ± SE (n = 5–7). Spearman’s *ρ* and the significance of a linear correlation through all points is shown (*** = *p* < 0.001).

**Figure 6:**
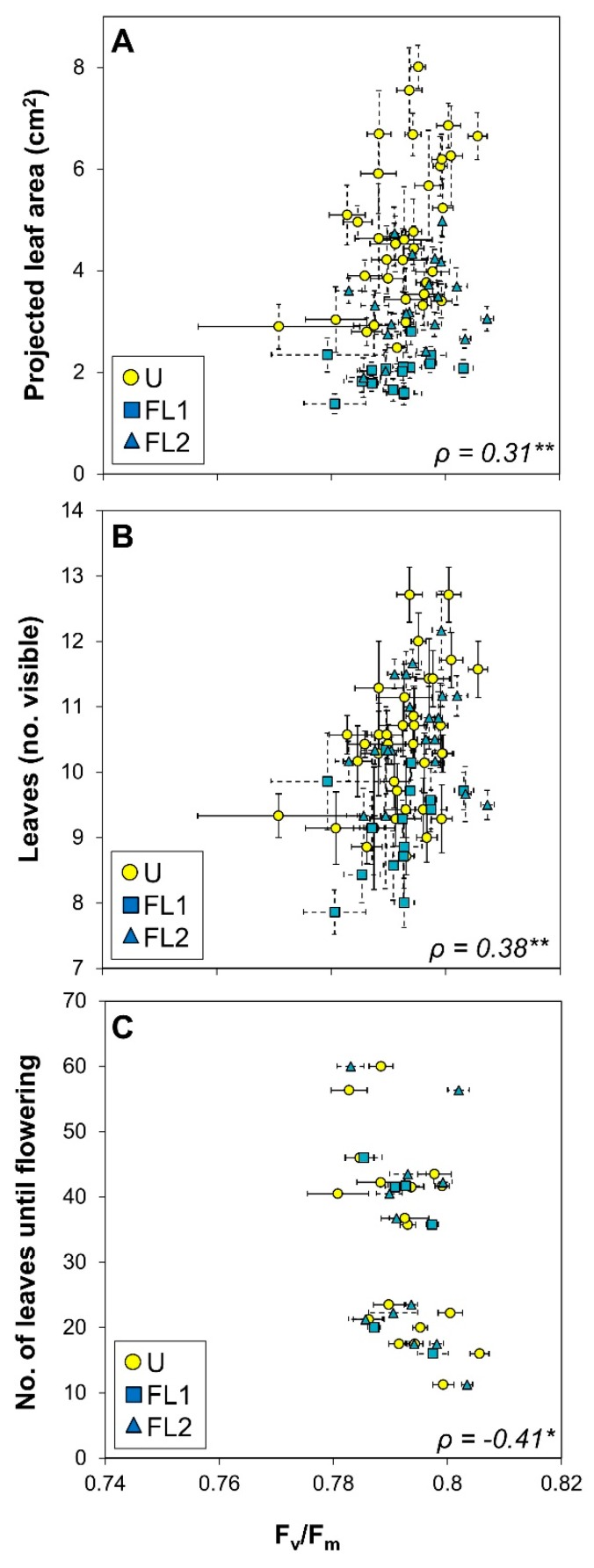
Relationships between photosystem II maximum quantum efficiency (F_v_/F_m_) and (**A**) projected leaf area, (**B**) the number of visible leaves and (**C**) the number of leaves required until flowering, in 36 Arabidopsis accessions acclimated to uniform (U) and fluctuating light intensities (FL). Data from plants grown under FL are sorted by experiment 1 (squares) and experiment 2 (triangles). Averages ± SE (n = 5–7). Spearman’s *ρ* and the significance of a linear correlation through all points is shown (** = *p* < 0.01, * = *p* < 0.05).

For a more detailed view of the interrelations between chlorophyll *a* fluorescence, growth, and development data, we constructed a correlation matrix between the average values for each accession and the measured variable, and we included latitude of origin and the number of leaves formed until flowering (Table 1). Strong correlations between most chlorophyll *a* fluorescence parameters (except for the relationship between NPQ and F_v_/F_m_) and all growth and developmental parameters were found (Table 2). Average leaf size correlated strongly and positively with Φ_PSII_, and negatively with NPQ (Figure 5), suggesting that light use efficiency had positive effects on leaf growth. These correlations are especially apparent for plants grown under U (yellow symbols in Figure 5). Both projected leaf area and the number of visible leaves correlated positively with F_v_/F_m_ (Figure 6A,B). Also, F_v_/F_m_ correlated negatively with the number of leaves formed until flowering (Figure 6C).

Next, we tested whether response ratios of parameters derived from FL over U grown plants (FL/U) correlated at the trait level, as well as with latitude of origin and number of leaves until flowering (Table 3). This analysis showed a strong, positive correlation between ΔΦ_PSII_ and ΔF_v_/F_m_ (Table 3), suggesting that accessions with a strong reduction in Φ_PSII_ also showed a stronger reduction in F_v_/F_m_ under FL. As might be expected, the response ratio of projected leaf area correlated strongly and positively with the response ratios of leaf number and leaf size (Table 3). We tested whether response ratios derived from either FL experiment 1 or FL experiment 2 yielded similar results, by repeating the same correlation analysis as shown in Table 3 for these two subsets of data (Tables S1 and S2). Both subsets yielded highly similar correlation coefficients, which themselves showed a strong linear correlation (Figure S1, *p* < 0.001). Correlation coefficients from each FL experiment subset also correlated strongly with those derived from the total dataset as shown in Table 3 (*p* < 0.001 in both cases, plots not shown). These results strongly suggest that the effects of fluctuating growth light on plants were repeatable within our experimental setup, and that hence similar conclusions can be drawn from both FL experiment 1 and FL experiment 2, further validating our findings.

**Figure 7:**
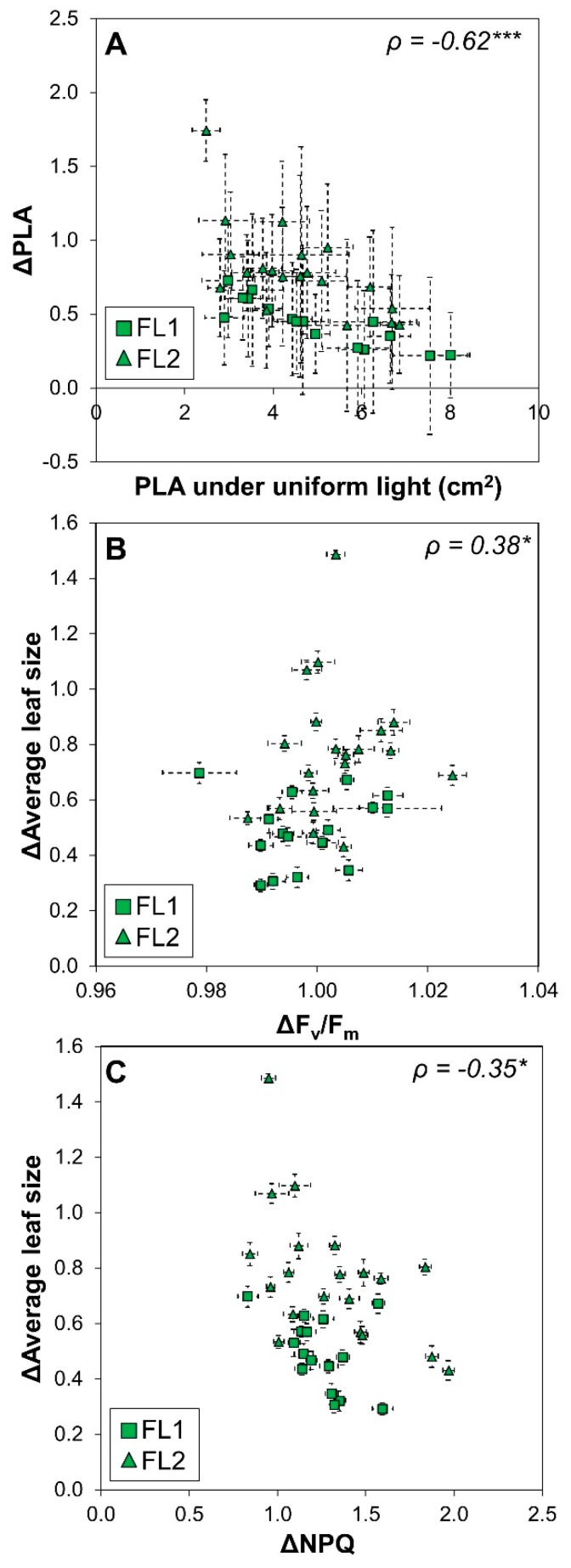
Changes in growth and chlorophyll *a* fluorescence in 36 Arabidopsis accessions grown under fluctuating compared to uniform light. (**A**) Relationship between projected leaf area (PLA) under uniform light and the response ratio of PLA under fluctuating light divided by PLA under uniform light (Δ = FL/U), (**B**) relationship between the response ratio of average leaf size and the response ratio of photosystem II maximum quantum efficiency (ΔF_v_/F_m_), and (**C**) relationship between the response ratio of average leaf size and the response ratio of non-photochemical quenching (ΔNPQ). Data are sorted by FL experiment 1 (squares) and FL experiment 2 (triangles). Averages ± SE (n = 5–7). Spearman’s *ρ* and the significance of a linear correlation through all points is shown (*** = *p* <0.001, * = *p*< 0.05).

**Figure 8:**
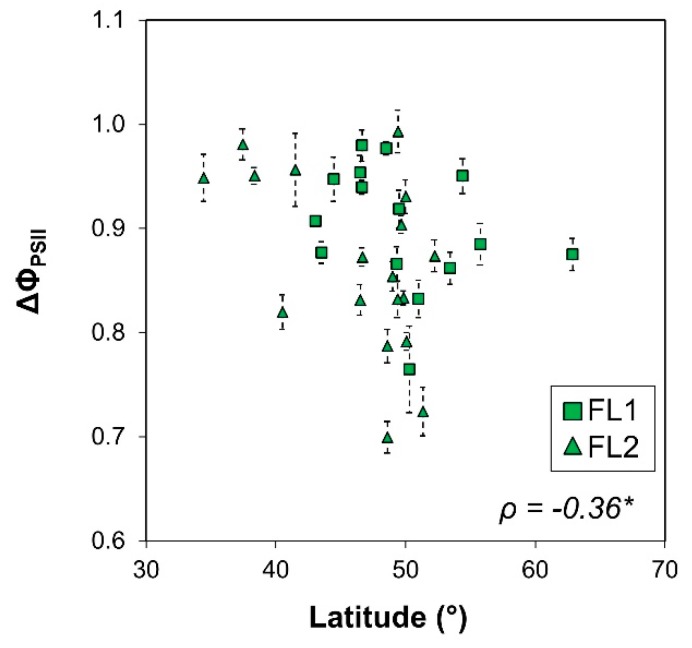
Relationship between latitude of origin of 36 Arabidopsis accessions and the response ratio in photosystem II operating efficiency (ΔΦ_PSII_) between plants grown under fluctuating light intensities (FL) divided by values from plants grown under uniform light intensities (Δ = FL/U). Data are sorted by FL experiment 1 (squares) and FL experiment 2 (triangles). Averages ± SE (n = 5–7). Spearman’s *ρ* and the significance of a linear correlation through all points is shown (*p* < 0.05).

The response ratio (FL/U) of PLA correlated strongly and negatively with PLA of plants grown under U (Figure 7A), suggesting that the reduction in PLA under FL was strongest in plants that showed high growth under U. The response ratio of average leaf size correlated positively with ΔF_v_/F_m_ and negatively with ΔNPQ (Figure 7B,C), again suggesting that leaf growth was directly related to rates of photoprotection and photoinhibition. Interestingly, the latitude of origin correlated negatively with ΔΦ_PSII_ (Figure 8), revealing a trend for photosynthesis of accessions collected further north on the globe to be more negatively affected by FL. Lastly, both the response ratio of projected leaf area and of number of visible leaves correlated positively with ΔF_v_/F_m_, but this correlation was less meaningful given the large uncertainty around the mean for values of single accessions (Figure S2).

## 3. Discussion

As photoautotrophs, plants interact with light in a direct manner. While our knowledge on rapid responses to changes in light intensity in the range of seconds to minutes is well advanced of plants grown under uniform light regimes (U; reviewed in [12,21,22,23]), less is known about the long-term response, i.e., acclimation occurring within days to fluctuating light (FL; [1,2]), and how this affects short-term responses to FL [2]. Here, we found that PLA and Φ_PSII_ generally decrease, NPQ generally increases, and F_v_/F_m_ as well as number of leaves per plant generally remain unchanged, when plants are grown under FL compared to U. This, together with the large phenotypic variation observed, broadly confirms our hypotheses. However, we acknowledge that because (i) plants grown under U and FL received different light sums and day lengths during the first 14 days after sowing and (ii) the FL experiment was run twice, with different groups of accessions each, further experimental work should be conducted to confirm the robustness of these results. Additionally, it will be desirable to increase the number of accessions for future analyses.

### 3.1. Fluctuating Light Reduces Visible Leaf Area Most Strongly in Plants with High PLA Under Uniform Light

Most (61%) accessions showed a reduction in projected leaf area under FL, and this reduction was substantial (36% reduction in PLA across accessions). This compares well to previous studies [7,8,9] in which a reduction of 30–58% in biomass across several FL regimes and species was shown. In our study, accessions with the largest PLA under U showed a much smaller PLA and a reduced number of leaves under FL, e.g., Fei-0, Amel-1, Col-0, and Gel-0 (Figure 2). That these accessions showed high PLA and leaf number in U suggests that they were not generally restricted in their capacity for growth. That these accessions had a decreased PLA and number of leaves under FL suggests that reduced photosynthesis under FL is the primary cause for the reduction in these two growth proxies. Here, we acknowledge that possible effects of FL on leaf angles and leaf thickness could be confounding factors when trying to correlate PLA with biomass, the direct read out for growth capacity. However, the decrease in PLA correlates with lower number of leaves, together suggesting that growth is negatively affected in FL. In nature, the reverse is often the case, i.e., plant growth rate constrains photosynthesis, mostly due to suboptimal temperatures and/or nutrient or water availability [24]. In our experiment, both scenarios of temperature- or nutrient-constrained growth seem unlikely for most accessions, as there was a strong negative correlation between PLA of plants grown under U and the response ratio of the same trait (Figure 7A). This correlation suggests that “fast growers” under U showed strong reductions under FL, and that consequently under U they were not restricted in their rate of leaf area expansion by factors other than those directly related to photosynthesis. Conversely, for most accessions that showed low PLA under U (Hs-0, Cen-0, Pla-0, Tsu-0; Figure 2), PLA, leaf number as well as Φ_PSII_ and NPQ were less strongly affected by FL (Figure 4A).

### 3.2. Large Natural Genetic Variation for PLA and Chlorophyll a Fluorescence Traits 

The coefficient of variation in accession-specific phenotypes ranged from 9 to 42%, suggesting that there was significant natural genetic variation (Figure 1, Figure 2, Figure 5, and Figure 6). Also, a large spread of accessions along principal components in the PCA was shown (Figure 3), suggesting that instead of distinct groups there was a continuum of responses. A similar range of genetic variation for traits related to photosynthesis and growth was previously found in populations of Arabidopsis [4,5], wheat [25,26] and rice [27]. The only exception to this observation was dark-adapted F_v_/F_m_ with a CV of only 0.8%, suggesting that it was not strongly affected by either treatment or accession. This is in agreement with previous data from Arabidopsis [4] and rice [27], in which the photosynthetic trait with the smallest genetic variation was F_v_/F_m_, but other traits varied considerably. The range in Φ_PSII_ values observed here (0.42–0.66; Figure 5A) compares very well to data reported by van Rooijen et al. [5], a study which in a larger panel of Arabidopsis accessions found a spread of 0.47–0.66 for Φ_PSII_ when this was determined in plants acclimated to and measured at 100 μmol m^−2^ s^−1^.

### 3.3. Correlations between Growth and Fluorescence: A Case for Rapid Phenotyping

Our data showed many interrelations between chlorophyll *a* fluorescence and growth-related traits (PLA, no. of visible leaves, average leaf size), suggesting that light use efficiency of photosystem II electron transport generally correlated with growth (Table 2 and Table 3; Figure 5, Figure 6 and Figure 7). Also, a strong negative and highly significant correlation (*p* < 0.001) between Φ_PSII_ and NPQ across treatments and accessions supports a link between photosynthetic efficiency and photoprotection (Table 2). This is remarkable, as this link is not often apparent at the relatively low light intensities that these measurements were conducted at (90 μmol m^−2^ s^−1^). 

Our data again emphasize chlorophyll *a* fluorescence imaging as a powerful tool for rapid plant phenotyping, enabling the analysis of photosynthesis-related traits in many accessions under multiple (and sometimes rapidly changing) environmental conditions [6,28,29]. Rapid plant phenotyping aims to simultaneously and repeatedly determine a large number of traits on a large number of plants. To that end, several weighing and imaging tools exist to determine, e.g., whole shoot and root growth [30], plant architecture, relative or absolute transpiration rates, as well as leaf temperature, photosynthetic capacity, spectral absorptivity [31], thickness, pigmentation and sugar concentration [32]. Since chlorophyll *a* fluorescence is often closely related to actual photosynthesis rates, and since photosynthesis reacts in a highly sensitive manner to intrinsic and extrinsic (e.g., environmental) factors, chlorophyll *a* fluorescence is a great tool to determine differences in photosynthesis within plants, across plants, and over time.

### 3.4. Can Evolutionary Adaptation Explain the Phenotype of an Accession?

Adaptation to a specific ecological niche shapes an organism’s genome and its response to changes in its surroundings [33]. Correlation analysis revealed a greater reduction in Φ_PSII_ under FL the further north on the globe the accession had originated from (Figure 8). This finding could potentially be explained by the following factors, considering that the main phase of vegetative growth of most Arabidopsis accessions is in early spring: (i) lower temperatures with increasing latitude, thus, accessions from further north may have optimized their photosynthesis to lower temperatures than those in our experiment (16 °C night, 21 °C day); (ii) differences in day length, because changes in gene expression that drive acclimation in response to FL have been shown to intercept with the circadian clock [3]; and/ or (iii) light intensity and availability.

A negative correlation was also found between F_v_/F_m_ and number of leaves until flowering (Figure 7C). The number of leaves until flowering is an indicator for whether Arabidopsis accessions germinate in spring or whether they germinate in autumn and require a long cold period in winter (vernalization) before flowering. Thus, it is tempting to speculate that species which form many leaves before flowering germinate in autumn and are thus better adapted to photosynthesis under cold temperatures, as they overwinter. A decrease in F_v_/F_m_ is associated with sustained photoinhibitory quenching (qI) that accompanies PSII damage. It has been speculated that slowly reversible qI may have a photoprotective function under some conditions [34]. Thus, qI may be prematurely switched on in winter-grown accessions in order to protect against cold-stress. 

## 4. Materials and Methods

### 4.1. Plant Material, Growth Conditions and Treatments

Thirty-five accessions of *Arabidopsis thaliana* were randomly selected from a collection of 330 accessions, and Col-0 was additionally selected as a reference genotype (36 accessions were used in total) due to its use as a wildtype in many reverse genetics studies. Information on country of origin, longitude, and the number of leaves until flowering (Table 1) was accessed on the 1001 genomes website (https://1001genomes.org/; [35]). In some cases, the Google Maps Arabidopsis viewer was additionally used to locate an accession. Latitudes for Col-0, Ler-1, and Ws-0 were omitted from Table 1 and correlation analyses (see below) as these genotypes have been cultivated in laboratories for decades and may not anymore be representative of the original accessions. All accessions are from the Northern Hemisphere, 28 (78%) were initially collected in Europe and another eight in Asia and North America (Table 1).

Seeds were sown on substrate prepared for Arabidopsis (‘Arabidopsis substrate’; 70% white peat, 20% vermiculite, 10% sand; Stender, Schermbeck, Germany) which was enriched with 1 g L^−1^ each of two standard fertilizers: Osmocote Start^®^ (ICL Specialty Fertilizers, Tel Aviv, Israel; composition: 11% N, 11% P_2_O_5_, 17% K_2_O, 2% MgO, 0.38% Fe, 0.05% Mn, 0.01% B, 0.09% Cu, 0.009% Mo, 0.014% Zn) and Triabon^®^ (Combo Expert, Münster, Germany; composition: 16% N, 8% P_2_O_5_, 12% K_2_O, 4% MgO, 9% S, 0.02% B, 0.04% Cu, 0.1% Fe, 0.1% Mn, 0.02% Mo, 0.01% Zn; [36]). For the first 14 days, plants that were later used in the uniform light treatment were grown under a 16 h photoperiod, at ~150 µmol m^−2^ s^−1^ photosynthetically active radiation (PAR; 400–700 nm), while plants later used for the fluctuating light treatment were grown in a 12 h photoperiod, at 250 µmol m^−2^ s^−1^ PAR, for the first 14 days. This means that FL grown plants were initially exposed to a higher daily light integral than those in U (10.8 mol photons d^−1^ and 8.6 mol photons d^−1^, respectively). Then, single plants were placed in a 0.11 L pot containing Arabidopsis substrate, and exposed to the light treatments until they were 28 days old. Day/night temperatures and relative humidity were 20/16 °C and 60/75% in all cases (for light and temperature recordings in the growth chambers, see Figure S3). 

The treatments were as follows: uniform light (U) of 250 µmol m^−2^ s^−1^ PAR, and fluctuating light (FL) of alternating cycles of 900 µmol m^−2^ s^−1^ and 90 µmol m^−2^ s^−1^ PAR for one and four minutes, respectively (average light intensity: 252 µmol m^−2^ s^−1^ PAR). In both treatments, the photoperiod was 12 h, totaling 144 high/low light cycles in the FL treatment. Changes between the two light intensities in the FL experiment were very rapid and accurate (Figure S3). Three types of LED lamps (Roschwege, Greifenstein, Germany) were used in both treatments: white (LED-Star 2700 K 10 W), red (LED-Star DR 660 nm 5 W) and blue (LED-Star DB 460 nm 5 W). The output setpoints of all LED lamps were kept identical for any given light intensity, ensuring that there were no changes in light spectrum as light intensity changed. Accessions under the FL treatment were grown in two separate experiments: 20 accessions were grown in experiment 1, 15 different accessions were grown in experiment 2, and Col-0 was grown in both experiments (Table 1). Utmost care was taken that conditions in both FL experiments were identical. In the U treatment, all accessions were grown in one experiment. Plants were watered 2–3 times per week depending on substrate wetness to the touch (as per usual practice in the institute). To account for position effects in the climate chamber, plants were randomized during the treatment period.

### 4.2. Chlorophyll *a* Fluorescence

Images of chlorophyll *a* fluorescence values were obtained using the IMAG-MAX/L imaging PAM system (Heinz Walz GmbH, Effeltrich, Germany), under which six plants were measured simultaneously. Plants that had been light-adapted under growth conditions were first dark-adapted for ≥20 min, after which minimal (F_o_) and maximal (F_m_) chlorophyll *a* fluorescence emissions were measured. Plants were then adapted to growth light conditions (≥30 min) for complete photosynthetic induction, after which they were shade-adapted at 90 µmol m^−2^ s^−1^ PAR in the imaging PAM for four minutes before chlorophyll *a* fluorescence emission under actinic light (F_s_) and maximal fluorescence from the light-adapted leaf (F_m_’) were determined. Saturating beam duration was 0.7 s and saturating beam intensity was ~1300 µmol m^−2^ s^−1^ when determining F_m_ and ~2700 µmol m^−2^ s^−1^ in the case of F_m_’. All measurements were conducted between 8:30 h and 14:00 h (growth lights switched on at 7:00). 

From chlorophyll *a* fluorescence images, in ImagingWin (v2.47, Heinz Walz GmbH) four circular areas of interest (AOI) were selected per plant. In ImagingWin, AOI with several pre-defined diameters can be selected. AOI were chosen to cover as much total plant leaf area as possible while avoiding parts of the picture not covered by plant material; consequently, AOI were typically chosen to cover parts of the largest leaves of a plant (Figure S4A). From each area of interest, an average value of F_o_, F_s_, F_m_ and F_m_’ was obtained. The four values were later averaged to represent one biological replicate. Photosystem II maximum quantum efficiency was calculated as F_v_/F_m_ = (F_m_ − F_o_)/F_m_, photosystem II operating efficiency was calculated as Φ_PSII_ = (F_m_’ − F_s_)/ F_m_’, and non-photochemical quenching was calculated as NPQ = (F_m_ − F_m_’)/F_m_’.

### 4.3. Growth and Development

Projected leaf area (PLA; i.e., total visible leaf area) was used as a proxy for growth, while the number of visible leaves was used as a proxy for development. For both variables, images of F_m_, obtained with the imaging PAM, were used (Figure S4B). ImageJ (https://imagej.nih.gov/ij/) was used to calculate PLA from the number of pixels per plant; pot diameter was used to scale pixel number to actual plant size. Average leaf size was obtained by dividing PLA by the number of visible leaves.

### 4.4. Clustering of Accessions by Phenotypic Differences

Accessions were clustered into different categories depending on log−2-transformed values of the response ratio (ΔP), which was calculated for each parameter (P) as ΔP = PFL/PU, where PU and PFL are the average values of the parameter for each accession. Cluster 3.0 (http://www.falw.vu/~huik/cluster.htm) with Euclidean distance as similarity metric and average linkage was used. Based on this result, categories were manually adjusted to fit the specified criteria.

### 4.5. Principal Component Analysis

A principal component analysis (PCA) was performed on the log−2-transformed response ratios (ΔP) using the prcomp function as implemented in R (https://www.r-project.org/).

### 4.6. Genomic Distances

Genomic distances between accessions were computed as Kimura distances using the program dnadist (v3.698, [37]). Genomic sequences of all accessions were taken as those composed of the 178,083 sites that were detected polymorphic in at least one of the 36 accessions. Original sequences were taken from [38] with exception of the sequence information of accession PHW-34, which was not included in the set, and was taken from [35].

### 4.7. Statistical Analysis

Means of each accession grown under the two treatments were compared using the nonparametric Wilcoxon’s rank-sum test, and *p*-values were adjusted for multiple comparisons using the Benjamini-Hochberg procedure [39]. Correlation analysis was performed on average responses per accession using Spearman’s rank correlation coefficient analysis. When correlation analysis was performed on response ratios, log−2 transformed values were used. Data were analyzed in R, using the packages ‘ggplot2’ by Hadley Wickham, ‘Hmisc’ by Frank Harrell and ‘ggpubr’ by Alboudakel Kassambra. The number of biological replicates per genotype and experiment was 5–7. 

## Figures and Tables

**Table 1:** Full names and abbreviations (as used in the figures) of Arabidopsis accessions. Accessions are sorted by the fluctuating light (FL) experiment they were used in. Information on country of origin, latitude, and number of leaves at flowering was accessed on the 1001 genomes website (https://1001genomes.org/). nd = not determined.

Experiment	Name	Abbreviation	Country of Origin	Latitude	#Leaves Flowering
1	Be-1	Be	Germany	49.68	nd
1	Cen-0	Cen	France	49.00	nd
1 & 2	Col-0	Col	USA	nd	16.00
1	Da(1)-12	Da	Czech Republic	49.85	17.50
1	DraIV6-16	Dra	Czech Republic	49.41	nd
1	Fei-0	Fei	Portugal	40.50	22.25
1	Ge-0	Ge0	Switzerland	46.50	60.00
1	Hn-0	Hn	Germany	51.35	21.25
1	Hs-0	Hs	Germany	52.24	17.50
1	Kelsterbach-2	Kel	Germany	50.07	nd
1	Ler-1	Ler	Germany	nd	11.25
1	Mnz-0	Mnz	Germany	50.00	23.50
1	PHW-34	P34	France	48.61	43.50
1	PHW-37	P37	France	48.61	nd
1	Pla-0	Pla	Spain	41.50	40.50
1	Sha	Sha	Tajikistan	38.35	nd
1	TOU-H-13	TOU	France	46.67	nd
1	Tsu-0	Tsu	Japan	34.43	36.75
1	Ws-0	Ws	Russia	nd	42.25
1	Yo-0	Yo	USA	37.45	56.33
1	ZdrI2-24	Zdr	Czech Republic	49.39	nd
2	Amel-1	Ame	Netherlands	53.45	41.50
2	Db-0	Db	Germany	50.31	nd
2	Ge-1	Ge1	Switzerland	46.50	nd
2	Gel-1	Gel	Netherlands	51.02	20.0
2	HSm	HSm	Czech Republic	49.33	41.66
2	Kin-0	Kin	USA	44.46	nd
2	LDV-58	LDV	France	48.52	nd
2	MNF-Che-2	MNF	USA	43.53	46.00
2	PAR-3	PAR	France	46.65	nd
2	Sapporo-0	Sap	Japan	43.06	nd
2	Ta-0	Ta	Czech Republic	49.50	35.75
2	Tad01	Tad	Sweden	62.87	nd
2	TDr-3	TDr	Sweden	55.77	nd
2	UKNW06-060	UKN	UK	54.40	nd
2	VOU-2	VOU	France	46.65	nd

**Table 2:** Correlation matrix for traits observed in plants grown under uniform and fluctuating light. Blue colored backgrounds indicate a positive correlation, red indicates negative; the more strongly colored the background, the steeper the slope of the correlation. Statistically significant correlations (*p* < 0.05) are marked in bold. Numbers indicate Spearman’s *ρ*, stars indicate the significance of the correlation, as: *** = *p* < 0.001, ** = *p* < 0.01 and * = *p* < 0.05 (n = 15–72). Lat., latitude of origin (°), #leaves flowering, number of leaves at flowering.

Trait	#Leaves at Flowering	Φ_PSII_	NPQ	F_v_/F_m_	PLA	#Leaves	Leaf Size
**Lat.**	−0.51	−0.03	−0.09	−0.05	−0.18	−0.16	−0.17
**#leaves at flowering**		−0.13	0.16	−0.41 *	0.16	0.10	0.10
**Φ_PSII_**			−0.64 ***	0.38 ***	0.52 ***	0.12	0.58 ***
**NPQ**				0.00	−0.41 ***	−0.08	−0.47 ***
**F_v_/F_m_**					0.31 **	0.38 **	0.27 *
**PLA**						0.72 ***	0.98 ***
**#leaves**							0.59 ***

**Table 3:** Correlation matrix for response ratio in traits under fluctuating light divided by those under uniform light (Δ = FL/U). Blue colored backgrounds indicate a positive correlation, red indicates negative; the more strongly colored the background, the steeper the slope of the correlation. Statistically significant correlations (*p* < 0.05) are marked in bold. Numbers indicate Spearman’s *ρ*, stars indicate the significance of the correlation, as: *** = *p* < 0.001, ** = *p* < 0.01 and * = *p* < 0.05 (n = 15–36). Lat., latitude of origin (°), #leaves flowering, number of leaves at flowering.

Trait	#Leaves Flowering	ΔΦ_PSII_	ΔNPQ	ΔF_v_/F_m_	ΔPLA	Δ#Leaves	ΔLeaf Size
**Lat.**	−0.51	−0.36 *	0.04	−0.03	−0.17	−0.07	−0.16
**#leaves at flowering**		0.07	0.05	0.13	−0.01	−0.01	0.06
**ΔΦ_PSII_**			−0.16	0.57 ***	0.17	0.16	0.14
**ΔNPQ**				0.05	−0.29	−0.19	−0.35 *
**ΔF_v_/F_m_**					0.42 *	0.48 **	0.38 *
**ΔPLA**						0.90 ***	0.98 ***
**Δ#leaves**							0.84 ***

## Supplementary Materials

The following is available online at https://www.mdpi.com/2223-7747/9/3/316/s1, Table S1: Correlation matrix for response ratio in traits under fluctuating light divided by those under uniform light (Δ = FL/U), in FL experiment 1, Table S2: Correlation matrix for response ratio in traits under fluctuating light divided by those under uniform light (Δ = FL/U), in FL experiment 2, Figure S1: Relationship between correlation coefficients shown in Table S1 (FL 1) vs. correlation coefficients in Table S2, Figure S2: Relationships between the response ratio of dark-adapted F_v_/F_m_ under fluctuating light divided by PLA under uniform light (Δ = FL/U) and A) the response ratio of projected leaf area as well as B) the response ratio of the number of visible leaves, Figure S3: Temperature (°C, left, red) and light intensity (µmol m^-2^ s^-1^, right, blue) measured in climate chamber for U (**A**) and FL (B,C) over the course of a day (A,B) and for 1 h (C) logged at 30 s interval, Figure S4: Data acquisition from chlorophyll a fluorescence pictures.

## References

[1] Alter P., Dreissen A., Luo F.L., Matsubara S. (2012). Acclimatory responses of Arabidopsis to fluctuating light environment: Comparison of different sunfleck regimes and accessions. Photosynth. Res..

[2] Matthews J.S.A., Vialet-Chabrand S., Lawson T. (2018). Acclimation to fluctuating light impacts the rapidity of response and diurnal rhythm of stomatal conductance. Plant Physiol..

[3] Schneider T., Bolger A., Zeier J., Preiskowski S., Benes V., Trenkamp S., Usadel B., Farré E.M., Matsubara S. (2019). Fluctuating light interacts with time of day and leaf development stage to reprogram gene expression. Plant Physiol..

[4] Rooijen R.V., Aarts M.G.M., Harbinson J. (2015). Natural genetic variation for acclimation of photosynthetic light use efficiency to growth irradiance in Arabidopsis. Plant Physiol..

[5] Van Rooijen R., Kruijer W., Boesten R., Van Eeuwijk F.A., Harbinson J., Aarts M.G.M. (2017). Natural variation of YELLOW SEEDLING1 affects photosynthetic acclimation of Arabidopsis thaliana. Nat. Commun..

[6] Cruz J.A., Savage L.J., Zegarac R., Hall C.C., Satoh-Cruz M., Davis G.A., Kovac W.K., Chen J., Kramer D.M. (2016). Dynamic Environmental Photosynthetic Imaging Reveals Emergent Phenotypes. Cell Syst..

[7] Vialet-Chabrand S., Matthews J.S.A., Simkin A.J., Raines C.A., Lawson T. (2017). Importance of fluctuations in light on plant photosynthetic acclimation. Plant Physiol..

[8] Kubásek J., Urban O., Šantrůček J. (2013). C4 plants use fluctuating light less efficiently than do C3 plants: A study of growth, photosynthesis and carbon isotope discrimination. Physiol. Plant..

[9] Leakey A.D.B., Press M.C., Scholes J.D., Watling J.R. (2002). Relative enhancement of photosynthesis and growth at elevated CO2 is greater under sunflecks than uniform irradiance in a tropical rain forest tree seedling. Plant, Cell Environ..

[10] Vaseghi M.J., Chibani K., Telman W., Liebthal M.F., Gerken M., Schnitzer H., Mueller S.M., Dietz K.J. (2018). The chloroplast 2-cysteine peroxiredoxin functions as thioredoxin oxidase in redox regulation of chloroplast metabolism. Elife.

[11] Kaiser E., Matsubara S., Harbinson J., Heuvelink E., Marcelis L.F.M. (2018). Acclimation of photosynthesis to lightflecks in tomato leaves: Interaction with progressive shading in a growing canopy. Physiol. Plant..

[12] Kaiser E., Galvis V.C., Armbruster U. (2019). Efficient photosynthesis in dynamic light environments: A chloroplast’ s perspective. Biochem. J..

[13] Pearcy R.W., Krall J.P., Sassenrath-Cole G.F., Baker N.R. (1996). Photosynthesis in fluctuating light environments. Photosynthesis and the Environment.

[14] Zhu X.G., Ort D.R., Whitmarsh J., Long S.P. (2004). The slow reversibility of photosystem II thermal energy dissipation on transfer from high to low light may cause large losses in carbon gain by crop canopies: A theoretical analysis. J. Exp. Bot..

[15] Suorsa M., Järvi S., Grieco M., Nurmi M., Pietrzykowska M., Rantala M., Kangasjärvi S., Paakkarinen V., Tikkanen M., Jansson S. (2012). PROTON GRADIENT REGULATION5 is essential for proper acclimation of Arabidopsis photosystem I to naturally and artificially fluctuating light conditions. Plant Cell.

[16] Armbruster U., Carrillo L.R., Venema K., Pavlovic L., Schmidtmann E., Kornfeld A., Jahns P., Berry J.A., Kramer D.M., Jonikas M.C. (2014). Ion antiport accelerates photosynthetic acclimation in fluctuating light environments. Nat. Commun..

[17] Takahashi S., Badger M.R. (2011). Photoprotection in plants: A new light on photosystem II damage. Trends Plant Sci..

[18] Kanazawa A., Kramer D.M. (2002). In vivo modulation of nonphotochemical exciton quenching (NPQ) by regulation of the chloroplast ATP synthase. PNAS.

[19] Armbruster U., Leonelli L., Galvis V.C., Strand D., Quinn E.H., Jonikas M.C., Niyogi K.K. (2016). Regulation and levels of the thylakoid K+/H+ antiporter KEA3 shape the dynamic response of photosynthesis in fluctuating light. Plant Cell Physiol..

[20] Caliandro R., Nagel K.A., Kastenholz B., Bassi R., Li Z., Niyogi K.K., Pogson B.J., Schurr U., Matsubara S. (2013). Effects of altered α- and β-branch carotenoid biosynthesis on photoprotection and whole-plant acclimation of Arabidopsis to photo-oxidative stress. Plant, Cell Environ..

[21] Kaiser E., Morales A., Harbinson J., Kromdijk J., Heuvelink E., Marcelis L.F.M. (2015). Dynamic photosynthesis in different environmental conditions. J. Exp. Bot..

[22] Kaiser E., Morales A., Harbinson J. (2018). Fluctuating light takes crop photosynthesis on a rollercoaster ride. Plant Physiol..

[23] Armbruster U., Correa Galvis V., Kunz H.H., Strand D.D. (2017). The regulation of the chloroplast proton motive force plays a key role for photosynthesis in fluctuating light. Curr. Opin. Plant Biol..

[24] Körner C. (2015). Paradigm shift in plant growth control. Curr. Opin. Plant Biol..

[25] Driever S.M., Lawson T., Andralojc P.J., Raines C.A., Parry M.A.J. (2014). Natural variation in photosynthetic capacity, growth, and yield in 64 field-grown wheat genotypes. J. Exp. Bot..

[26] Carmo-Silva E., Andralojc P.J., Scales J.C., Driever S.M., Mead A., Lawson T., Raines C.A., Parry M.A.J. (2017). Phenotyping of field-grown wheat in the UK highlights contribution of light response of photosynthesis and flag leaf longevity to grain yield. J. Exp. Bot..

[27] Qu M., Zheng G., Hamdani S., Essemine J., Song Q., Wang H., Chu C., Sirault X., Zhu X.G. (2017). Leaf photosynthetic parameters related to biomass accumulation in a global rice diversity survey. Plant Physiol..

[28] Murchie E.H., Kefauver S., Araus J.L., Muller O., Rascher U., Flood P.J., Lawson T. (2018). Measuring the dynamic photosynthome. Ann. Bot..

[29] van Bezouw R.F.H.M., Keurentjes J.J.B., Harbinson J., Aarts M.G.M. (2019). Converging phenomics and genomics to study natural variation in plant photosynthetic efficiency. Plant J..

[30] Fiorani F., Schurr U. (2013). Future Scenarios for Plant Phenotyping. Annu. Rev. Plant Biol..

[31] Dutta S., Cruz J.A., Jiao Y., Chen J., Kramer D.M., Osteryoung K.W. (2015). Non-invasive, whole-plant imaging of chloroplast movement and chlorophyll fluorescence reveals photosynthetic phenotypes independent of chloroplast photorelocation defects in chloroplast division mutants. Plant J..

[32] Yendrek C.R., Tomaz T., Montes C.M., Cao Y., Morse A.M., Brown P.J., McIntyre L.M., Leakey A.D.B., Ainsworth E.A. (2017). High-throughput phenotyping of maize leaf physiological and biochemical traits using hyperspectral reflectance. Plant Physiol..

[33] Hancock A.M., Brachi B., Faure N., Horton M.W., Jarymowycz L.B., Sperone F.G., Toomajian C., Roux F., Bergelson J. (2011). Adaptation to climate across the Arabidopsis thaliana genome. Science (80-. ).

[34] Lee H.Y., Wah S.C., Hong Y.-N. (1999). Photoinactivation of Photosystem II in leaves of Capsicum annuum. Photosynth. Res..

[35] (2016). Consortium, 1001 Genomes 1,135 Genomes Reveal the Global Pattern of Polymorphism in Arabidopsis thaliana. Cell.

[36] Köhl K., Tohge T., Schöttler M.A. (2017). Performance of Arabidopsis thaliana under different light qualities: Comparison of light-emitting diodes to fluorescent lamp. Funct. Plant Biol..

[37] Felsenstein J. (2019). PHYLIP (Phylogeny Inference Package), version 3.698.

[38] Horton M.W., Hancock A.M., Huang Y.S., Toomajian C., Atwell S., Auton A., Muliyati N.W., Platt A., Sperone F.G., Vilhjálmsson B.J. (2012). Genome-wide patterns of genetic variation in worldwide Arabidopsis thaliana accessions from the RegMap panel. Nat. Genet..

[39] Benjamini Y., Hochberg Y. (1995). Controlling the false discovery rate: A practical and powerful approach to multiple testing. J. R. Stat. Soc. Ser. B.

