# Supplementary material for: Growth under Fluctuating Light Reveals Large Trait Variation in a Panel of Arabidopsis Accessions
Source: Plants (Basel). 2020 Mar 3;9(3):316. doi: 10.3390/plants9030316 (PMC7154909; doi:10.3390/plants9030316)
Supplement: Supplementary file 1 [file plants-09-00316-s001.pdf]

plants

MDPI

Table S1. Correlation matrix for response ratio in traits under fluctuating light divided by those under uniform light (Δ = FL/U), in FL experiment 1. Blue colored backgrounds indicate a positive correlation, red indicates negative; the more strongly colored the background, the steeper the slope of the correlation. Statistically significant correlations ($p < 0.05$) are marked in bold. Numbers indicate Spearman's ρ, stars indicate the significance of the correlation, as: *** = $p < 0.001$, ** = $p < 0.01$ and * = $p < 0.05$ (n = 10–20). Lat., latitude of origin (°), #leaves flowering, number of leaves at flowering.

Trait	#leaves flowering	$\Delta\Phi_{PSII}$	ΔNPQ	$\Delta F_v/F_m$	ΔPLA	Δ#leaves	ΔLeaf size
**Lat.**	-0.58	-0.31	0.05	0.04	-0.16	-0.06	-0.1
**#leaves at flowering**		0.21	0.07	0	0.01	-0.11	0.03
$\Delta\Phi_{PSII}$			-0.28	**0.75*** **	**0.49***	**0.48***	0.44
**ΔNPQ**				-0.02	**-0.48***	**-0.47***	**-0.49***
$\Delta F_v/F_m$					0.3	**0.48***	0.22
**ΔPLA**						**0.84*** **	**0.98*** **
**Δ#leaves**							**0.77*** **

Table S2. Correlation matrix for response ratio in traits under fluctuating light divided by those under uniform light (Δ = FL/U), in FL experiment 2. Blue colored backgrounds indicate a positive correlation, red indicates negative; the more strongly colored the background, the steeper the slope of the correlation. Statistically significant correlations ($p < 0.05$) are marked in bold. Numbers indicate Spearman's ρ, stars indicate the significance of the correlation, as: *** = $p < 0.001$, ** = $p < 0.01$ and * = $p < 0.05$ (n = 5–16). Lat., latitude of origin (°), #leaves flowering, number of leaves at flowering.

Trait	#leaves flowering	$\Delta\Phi_{PSII}$	ΔNPQ	$\Delta F_v/F_m$	ΔPLA	Δ#leaves	ΔLeaf size
**Lat.**	-0.7	-0.39	0.12	0.11	-0.02	0.03	-0.02
**#leaves at flowering**		-0.2	-0.14	0.54	0.09	0.31	0.14
$\Delta\Phi_{PSII}$			-0.32	**0.51***	0.22	0.22	0.26
**ΔNPQ**				0.09	-0.35	-0.21	-0.49
$\Delta F_v/F_m$					0.3	0.24	0.28
**ΔPLA**						**0.9*** **	**0.97*** **
**Δ#leaves**							**0.84*** **

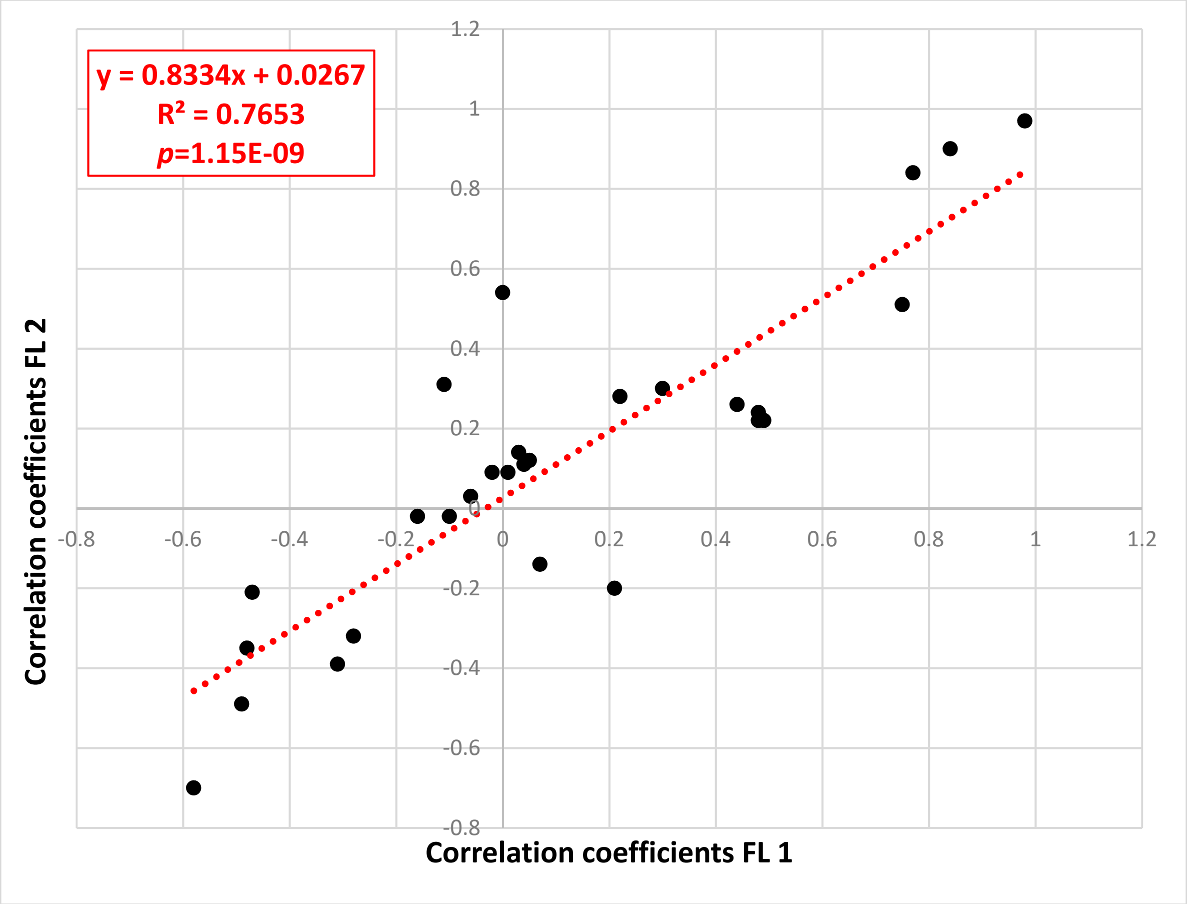

Figure S1. Relationship between correlation coefficients shown in Table S1 (FL 1) vs. correlation coefficients in Table S2 (FL 2). The correlation between both datasets (red line) is highly linear and highly significant, suggesting that similar conclusions can be drawn from both subsets of data.

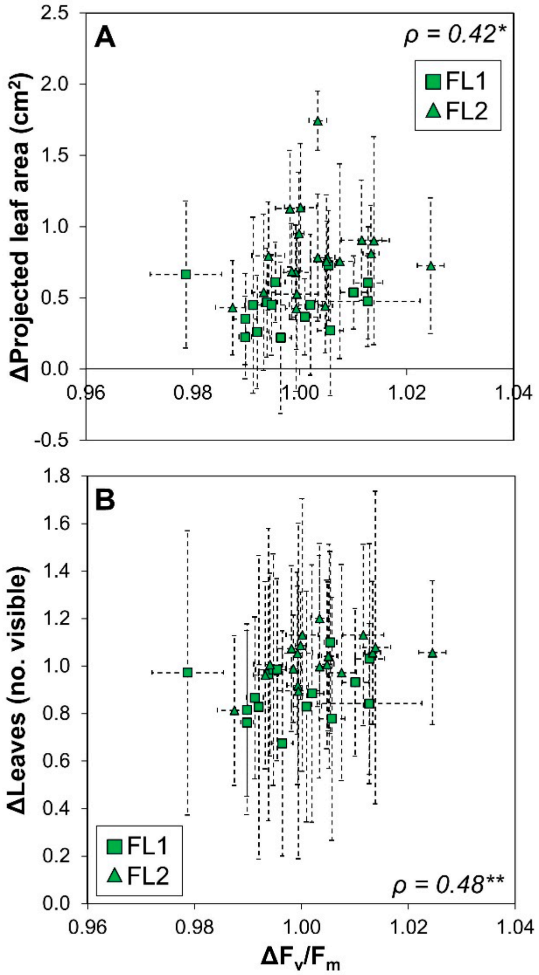

Figure S2. Relationships between the response ratio of dark-adapted F_v/F_m under fluctuating light divided by PLA under uniform light (Δ = FL/U) and A) the response ratio of projected leaf area as well as B) the response ratio of the number of visible leaves. Data are sorted by FL experiment 1 (squares) and FL experiment 2 (triangles). Averages ± SE (n=5–7). Spearman's ϱ and the significance of a linear correlation through all points is shown (** = p<0.01, * = p<0.05).

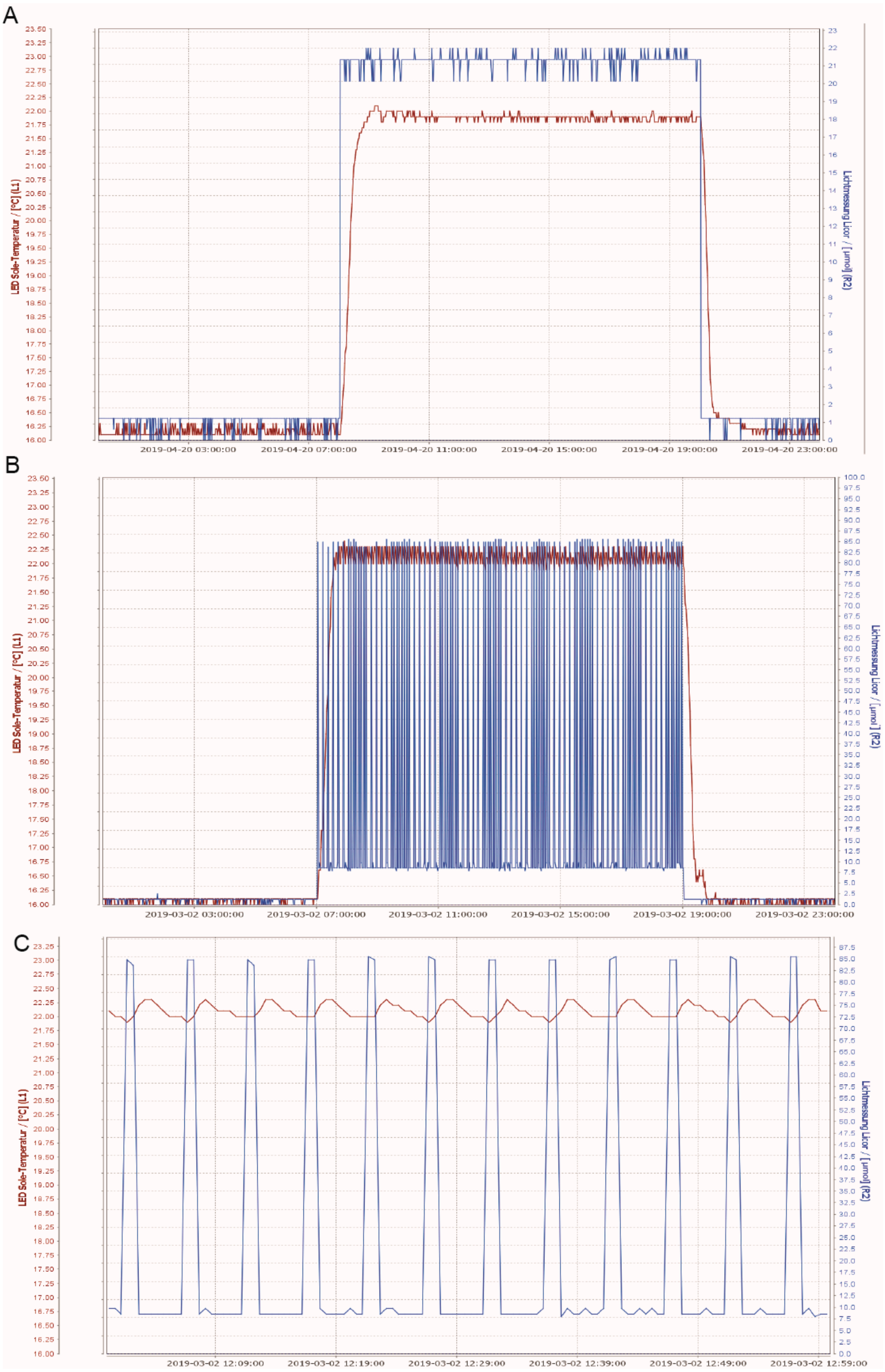

Figure S3. Temperature (°C, left, red) and light intensity (µmol m^{-2} s^{-1}, right, blue) measured in climate chamber for U (**A**) and FL (B,C) over the course of a day (A,B) and for 1 h (C) logged at 30 s interval. Note that the light sensor was placed perpendicular to the LEDs below the plants and thus did not measure the actual light intensity that plants received. The figure shows that fluctuations in light intensity only cause minor fluctuations in chamber temperature (< 0.5 °C).

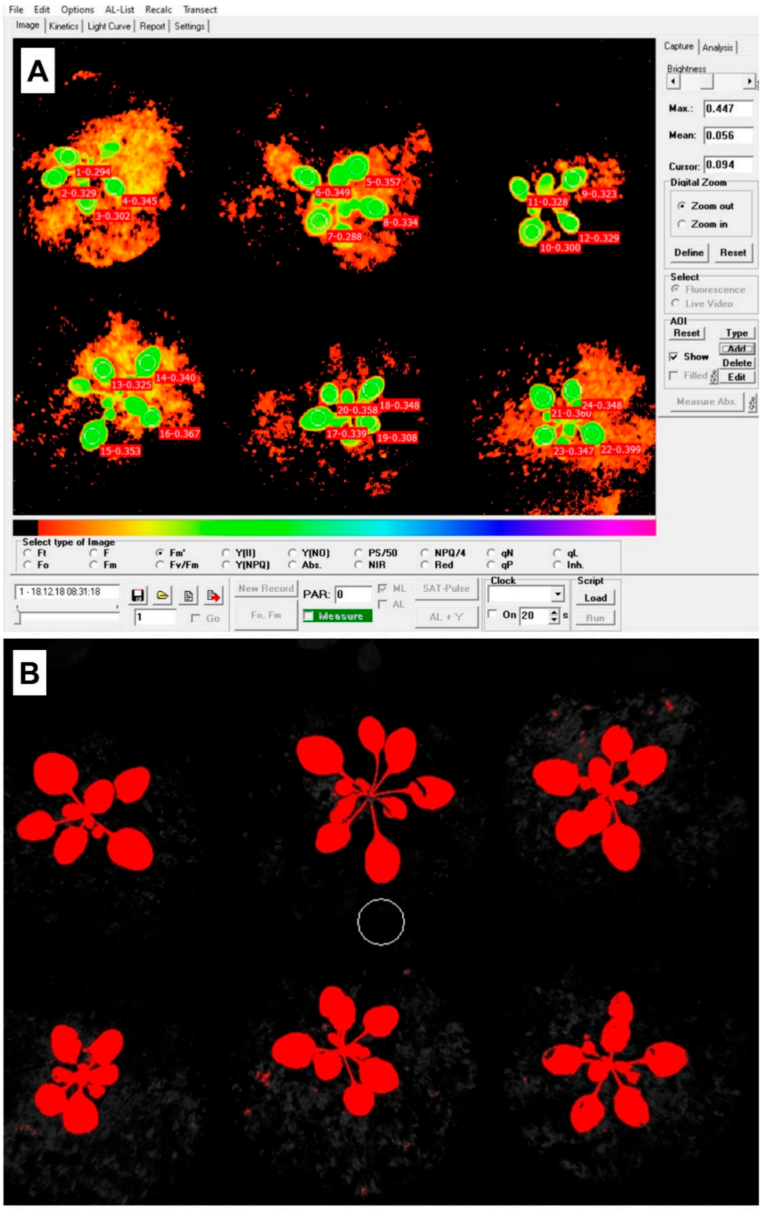

Figure 4. Data acquisition from chlorophyll a fluorescence pictures. **A**) Example of the selection of four areas of interest (AOI) per plant for determination of average chlorophyll fluorescence parameters, in ImagingWin (v2.47, Heinz Walz GmbH, Effeltrich, Germany). AOI were determined using F_m', since its value in plants was clearly different to the background value of the substrate. **B**) Example of picture used for determination of projected leaf area (PLA), based on F_m. Pictures for PLA determination were first generated in ImagingWin, and then imported to ImageJ.
